# Luminescent Oxygen Sensor with Self-Sterilization Properties Based on Platinum(II)octaethylporphyrin in Polymeric Nanofibers

**DOI:** 10.1021/acsmaterialsau.4c00137

**Published:** 2025-01-01

**Authors:** Pavel Ludačka, Vojtěch Liška, Jan Sýkora, Pavel Kubát, Jiří Mosinger

**Affiliations:** † Faculty of Science, Charles University, Hlavova 2030, Prague 2 128 43, Czech Republic; ‡ J. Heyrovský Institute of Physical Chemistry of the Czech Academy of Sciences, Dolejškova 3, Prague 8 182 23, Czech Republic

**Keywords:** oxygen sensing, luminescence, polycaprolactone, nanofibers, antibacterial

## Abstract

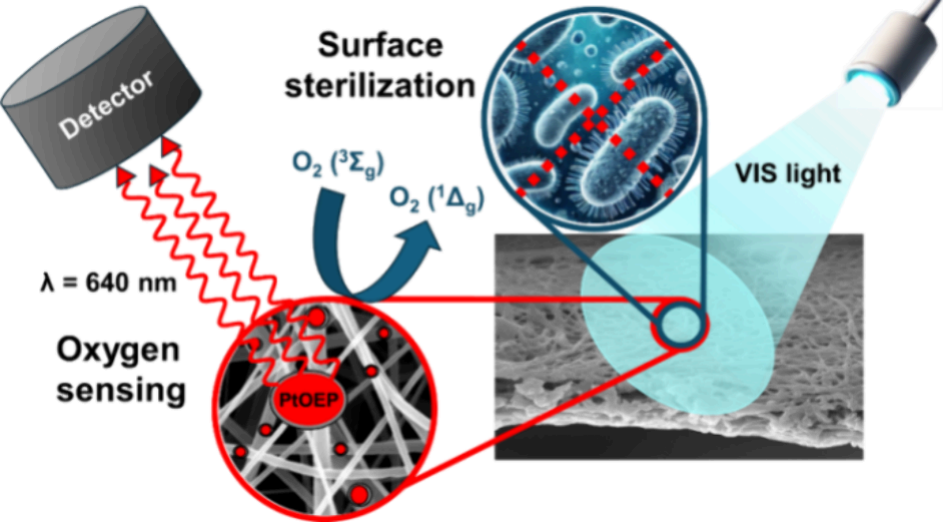

Optical sensors based on the quenching of the luminescence of platinum(II)octaethylporphyrin (PtOEP) encapsulated in nanofiber polymeric membranes were prepared by electrospinning. The samples were characterized using scanning electron microscopy, confocal luminescence microscopy, absorption spectroscopy, and steady-state and time-resolved luminescence techniques. The properties of the sensors were changed by the selection of different polymeric membranes using polycaprolactone, polystyrene, polyurethane Tecophilic, and poly(vinylidene fluoride-*co*-hexafluoropropylene) polymers. Among them, biodegradable and biocompatible sensors prepared from polycaprolactone with a high oxygen diffusion coefficient exhibited a fast response time (0.37 s), recovery time (0.58 s), high sensitivity (maximum *I*_*0*_*/I* ratio = 52), reversible luminescent response, and linear Stern–Volmer quenching over the whole range of oxygen contents in both the gas atmosphere and aqueous media. Moreover, the proposed sensors exhibited high antibacterial properties, resulting in self-sterilization character of the membrane surface due to the photogeneration of singlet oxygen. This dual character can find application in the biomedical field, where both properties (oxygen sensing and self-sterilization) can be acquired from the same material.

## Introduction

Oxygen plays a fundamental role in respiration and metabolism, and quantifying oxygen levels, especially at the microscale, is essential in many biological and medicinal applications.^1−5^ Conventional Clark electrodes are difficult to miniaturize and consume oxygen during measurements.^6,7^ In contrast, optical oxygen sensors based on the quenching of luminescence intensity or lifetime to quantify oxygen levels^8^ have been developed and applied across many biological systems.^6,9,10^ Their advantages include a lack of oxygen consumption, noninvasive characteristics, and freedom from electrical interference.^11^

**Figure 1:**
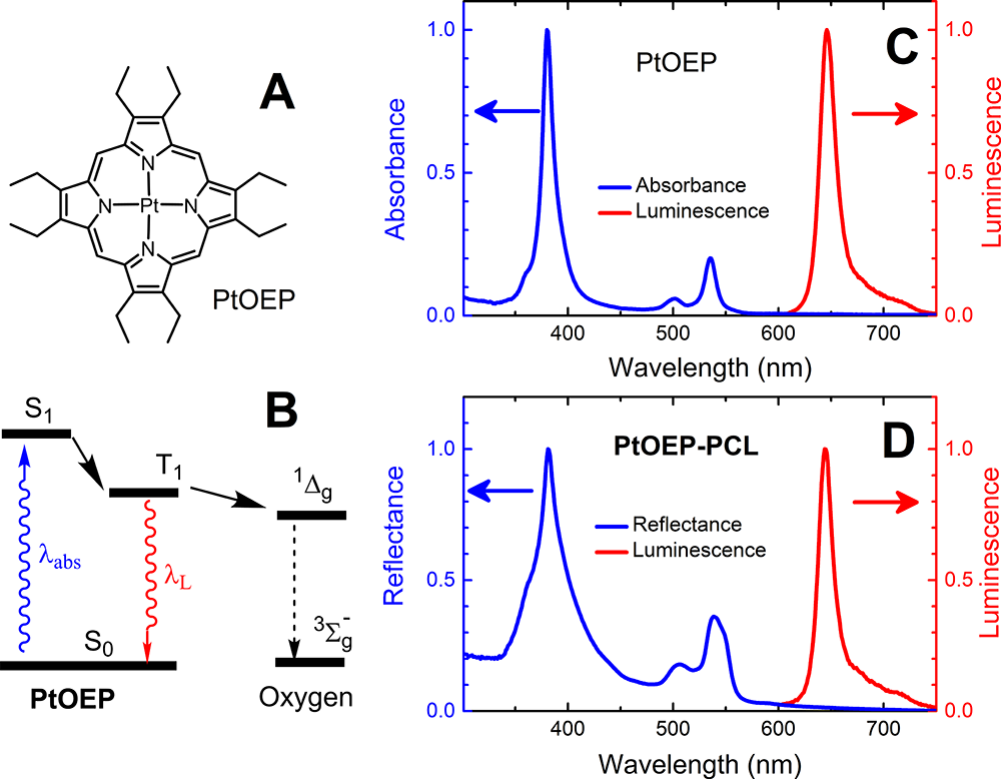
Structure of the PtOEP oxygen sensor (A), simplified Jablonski diagram illustrating PtOEP luminescence quenching by molecular oxygen (B). Absorption and luminescence spectra of PtOEP in solution (C). Reflection and luminescence spectra of PtOEP in PCL nanofibers (**PtOEP-PCL,** D).

The most commonly used luminescent molecules are platinum(II) and palladium(II) tetrakis(pentafluorophe nyl)porphyrin and tris(4,7-diphenyl-1,10-phenanthroline) ruthenium(II) dichloride. In addition to these luminescent molecules, platinum(II) octaethylporphyrin (PtOEP, Figure 1), which is usually present in different matrices, is typically used for oxygen sensing.^12−15^ PtOEP exhibits strong luminescence at room temperature with a quantum yield of approximately 0.4, a large Stokes shift, and a relatively long luminescence lifetime.^16^

Oxygen sensors are often incorporated into matrices with porous structures (silica gels, materials based on silica, aerogels, electrospun polymeric nanofibers, metal–organic frameworks, etc.)^17^ or nonporous structures (e.g., polymeric films).^9^ Most of the selected matrix materials exhibit high oxygen diffusion coefficients. The common feature of porous structures is relatively fast oxygen diffusion, which enhances the sensitivity of the sensors and improves the response and recovery times. The polymeric films show excellent performance and stability, but their sensitivity is still limited, as they exhibit relatively long response and recovery times and are not suitable for biological applications at microscale levels. Nanofiber materials could enable sensing of oxygen not only over large areas but also on a submicrometer scale using a small piece of the material or a single nanofiber.

Recently, electrospun nanofiber materials have been introduced as matrices for sensors.^18^ They combine favorable properties of both porous and stable polymeric films. Biocompatible polymers and copolymers with high diffusion coefficients have been used for electrospinning and fabrication of nanofiber materials.^19^ Xu et al.^8^ created nanofibers consisting of either a poly(ether sulfone) or a polysulfone core coated with a biocompatible polycaprolactone (PCL) shell. Oxygen-sensitive luminescent Pt(II) and Pd(II) *meso*-tetra(pentafluorophenyl) porphyrin were incorporated into the core with relatively low oxygen permeability but still exhibited excellent properties for oxygen sensing, including a fast response and recovery time. As noted in the study, the low oxygen permeability can be compensated for by other key features of the used polymer matrix/sensor molecules (probes), such as high brightness, low probe molecule aggregation in the polymer, optical clarity and ability to be used in the biomedical field.

To follow the same strategy, in this work, we incorporate a nonpolar PtOEP luminescent oxygen sensor directly into PCL nanofibers via a simple electrospinning process and compare its properties with those reported in the literature and with the properties of PtOEP dispersed in spinnable polystyrene (PS), poly(vinylidene fluoride-*co*-hexafluoropropylene (PVDFHFP), and Tecophilic (TECO) nanofibers, all of which have relatively high oxygen diffusion coefficients. In particular, PCL has a high oxygen diffusion coefficient (1.5 × 10^7^ cm^2^ s^–1^),^20^ is nontoxic and biodegradable, has low cost, has excellent mechanical properties, primarily tensile strength expressed as the Young’s modulus;^21^ additionally, PCL can be used in biomedical applications as an example of an adherent scaffold for tissue engineering.^22^

In addition to the excellent luminescence properties important for sensing, PtOEP in a competitive deactivation channel also acts as a photosensitizer (Figure 1B) that enables the formation of antibacterial singlet oxygen, O_2_(^1^Δ_g_), via energy transfer from an excited PtOEP to oxygen. The new functionality, i.e., the antibacterial character of the sensor via O_2_(^1^Δ_g_) photogeneration during sensing and/or vis irradiation, can suppress surface biofilm formation, which limits the biomedical application and the accessibility of the sensor to oxygen. The photogeneration of O_2_(^1^Δ_g_) and the corresponding light-triggered antibacterial properties of the sensor surface, which are important for its self-sterilization, were also evaluated in this study.

This paper is focused on nanofiber membranes with encapsulated PtOEP enabling a combination of oxygen sensing with antibacterial effect, the optimization of the polymeric material for the fabrication of nanofibers, description of the sensor behavior on a submicron scale, and experimental proof of antibacterial properties of the optimized material.

## Experimental Methods

### Chemicals

The platinum(II) complex of octaethylporphyrin (PtOEP), 5,10,15,20-tetraphenylporphin (TPP), polystyrene (*M*_w_ 192 000), poly(vinylidene fluoride-*co*-hexafluoropropylene) (*M*_w_ 400 000) and tetraethylammonium bromide (TEAB) were purchased from Sigma-Aldrich (USA), the Tecophilic HP-60D-60 from Lubrizol (USA), and the poly(ε-caprolacton) (*M*_w_ 80 000) CAPA 6800 from Ingevity (USA).

Tetrahydrofuran (THF), dimethylformamide (DMF), dichloromethane (DCM) and all inorganic salts, acids and hydroxides used for iodide detection solution preparation were purchased from Penta (Czech Republic). The chemicals used for bacterial testing, such as Triton X 100, ROTI CELL PBS, LB agar, and LB media, were obtained from Carl Roth GmbH + Co. KG (Germany), and the chemicals used for bacterial detection, namely, 5-bromo-4-chloro-3-indolyl β-D-galactopyranoside and isopropyl β-D-1-thiogalactopyranoside, were obtained from Sigma-Aldrich (USA).

### Preparation of Optical Oxygen Sensors Using Different Polymers

Optical oxygen sensors containing PtOEP dispersed in various polymeric nanofiber materials were prepared by electrospinning. The general procedure for preparing the materials was as follows: A mixture of 1 wt % PtOEP and 99 wt % polymer (PCL, PS, PVDFHFP, or TECO) were dissolved in spinning solvent(s) to prepare 6–10 wt % polymer solutions for the fabrication of photoactive nanofiber materials. The conductivity of the spinning solutions was enhanced in some cases by the addition of TEAB (0.13 wt %) in DMF. The solutions were electrospun via needle electrospinning technology^23^ at high voltage (12–22 kV), a current of 0.01–0.03 A and a flow rate of 1.0 mL/h for 15 min. The nanofiber materials were deposited on an Al foil covering the collecting electrode.

### Basic Characterization of the Materials

The morphology of the prepared materials was characterized by scanning electron microscopy (SEM) – using a Quanta 200 FEG scanning electron microscope (FEI, Czech Republic). The nanofiber diameters were measured by NIS Elements 4.0 image analysis software (Laboratory Imaging, Czech Republic). The area density was determined gravimetrically by weighing the area of the nanofiber material via an analytical balance (A&D GR 200). The average thickness of the materials was determined from 3 independent measurements via a Mitutoyo thickness gauge (Japan).

### Spectral and Photophysical Properties

The UV/vis absorption spectra were recorded via Unicam 340 and Varian 4000 spectrometers. The steady-state luminescence was measured with an Edinburgh Instruments FLS 980 spectrometer using a flow-through luminescence cell equipped with a commercial ISO OXY-2 oxygen sensor from Word Precision Instruments (Clark electrode) and connected to nitrogen and oxygen gas bottles. A sample (3 × 1 cm) of nanofiber material on a quartz glass support was diagonally placed in a cell such that the luminescence and oxygen content were measured by a Clark electrode at the same time. The different gas atmospheres and dissolved oxygen contents were maintained by the addition of nitrogen or oxygen. The response time and recovery time were measured in kinetic mode of the spectrometer after the fast change in the atmosphere in the flow-through cell as the time interval for reaching 95% of the maximum response.

Time-resolved luminescence experiments were performed in vacuum, air, and oxygen atmospheres at 24 °C with a Quantel Smart 450 Nd YAG laser (excitation wavelength 355 nm, fwhm ∼5 ns). The time-resolved luminescence of the materials at 640 nm was measured via an LKS 20 laser kinetic spectrometer (Applied Photophysics, UK) equipped with a Hamamatsu R928 photomultiplier. The materials were evacuated for at least 30 min by a rotary pump for measurements under vacuum.

### Confocal Luminescence Microscopy

Confocal luminescence microscopy was carried out on a custom-built confocal microscope. The excitation source (pulsed diode laser, PicoTa, Toptica, 535 nm controlled by Sepia II, Picoquant) was operated in the sequence of 20 subsequent laser pulses separated by 12.5 ns followed by 31.25 μs of the idle period. The excitation light was focused into the sample through a water immersion objective (Olympus, 1.2 NA, 60x). The excitation and emission light were separated by a dichroic mirror Z473/532 (Chroma), and the luminescence was further guided through a 50 μm pinhole into the detection channel (bandpass filter 646/42, MPD detector (PDM)). The detected photons were registered by the TCSPC module Hydraharp 400 (Picoquant) with an overall time span of 32.13 μs and a temporal resolution of 1 ns/channel.

### Photogeneration of Singlet Oxygen

The generation of O_2_(^1^Δ_g_) was confirmed by time-resolved near-infrared luminescence at 1270 nm after excitation by a Quantel Nd YAG laser (wavelength of 355 nm, pulse length of 5 ns).^24^ The amount of O_2_(^1^Δ_g_) released from the nanofibers was followed by chemical method based on the iodide detection solution described in our previous paper.^25^ Briefly, a 2 × 1 cm sample was mounted on quartz glass and irradiated in a cuvette with iodide detection solution by a 400 W solar simulator (Sol1A Newport, USA). The kinetics of the photogenerated triiodide visible at 351 nm was monitored and compared with that of control solutions of the same composition that were kept in the dark, solutions with 0.01 M sodium azide as a singlet oxygen quencher, and solutions of the iodide detection solution that were irradiated alone without a sample.

### Antibacterial Assays

To demonstrate the antibacterial effect, two different antibacterial assays were performed on *Escherichia coli* DH5α (Invitrogen, California, USA) with the plasmid pGEM11Z (Promega, Wisconsin, USA) bacterial strain. The X-Gal assay was purposely used for qualitative evaluation and visualization; the antibacterial assay with CFU counting was used to evaluate the antibacterial effect quantitatively.

For the X-Gal assay, the bacteria were allowed to grow at 37 °C until the *A*_600_ value reached approximately 1, after which the bacterial suspension was diluted 1000 times in PBS. The nanofiber samples (4 cm^2^) were placed on a sterile agar plate to retain moisture, and 20 μL of the diluted bacterial suspension was applied to the surface. The samples were subsequently irradiated with light from a 400 W solar simulator (Sol1A Newport, USA) with a 400 nm cutoff filter applied for 10 min from a distance of 33 cm or kept in the dark for the same duration as the controls. Following irradiation, 20 μL of X-Gal (20 mg/mL in 50% DMF) was added, followed by 20 μL of IPTG (23 mg/mL in H_2_O) after soaking. The plates were incubated for 20 h in the dark at 37 °C to allow individual bacteria to grow and form colonies, which were visualized as blue dots on the sample surface due to X-Gal cleavage.

A CFU counting antibacterial test was conducted to assess the efficacy of the prepared membrane in comparison to other commonly used photosensitizers with documented antibacterial activity. The tetraphenyl porphyrin (TPP) membrane was selected for this purpose. A 20-μL aliquot of 1000-fold diluted bacterial suspension of *Escherichia coli* in PBS with 0.2% Tween 80 (A_600_ ∼ 1.7 before dilution) was applied to the surface of the membranes (2 × 2 cm). The samples were exposed to a 400 W solar simulator (Sol1A Newport, USA) with a 400 nm cutoff filter on a wet cotton pad for 10 min or kept in the dark for the same period as the controls. The samples were subsequently vortexed vigorously for 60 s in 400 μL of PBS with 0.2% Tween 80. A total of 150 μL was taken from each sample and plated on clean agar plates in duplicate. The agar plates were incubated in the dark at 37 °C for 20 h to allow the growth of colonies. The agar plates were subsequently photographed, and the number of surviving colonies (colony-forming units, CFU) was subsequently quantified via image analysis using OpenCFU. The results shown are the average of three independent experiments.

To visualize the antibacterial effect on the surface of **PtOEP-PCL**, two samples were analyzed using SEM: one was exposed to a 400 W solar simulator light for 10 min and the other kept in dark. **PtOEP-PCL (**2.25 cm^2^) was inoculated with 200 μL of 200x diluted *Escherichia coli* suspension (A_600_ ∼ 1.5). The method of next fixation and SEM analysis were described in previous study.^26^

## Results and Discussion

The samples of polymeric nanofiber membranes without sensor molecules (**PCL, PS TECO, and PVDFHFP**), as well as the corresponding ones with encapsulated PtOEP sensor molecules (**PtOEP-PCL, PtOEP-PS, PtOEP-TECO, and PtOEP-PVDFHFP**), were prepared via an electrospinning process (see Experimental methods) and used as-prepared for this study.

### Characterization of Nanofiber Materials

**Figure 2:**
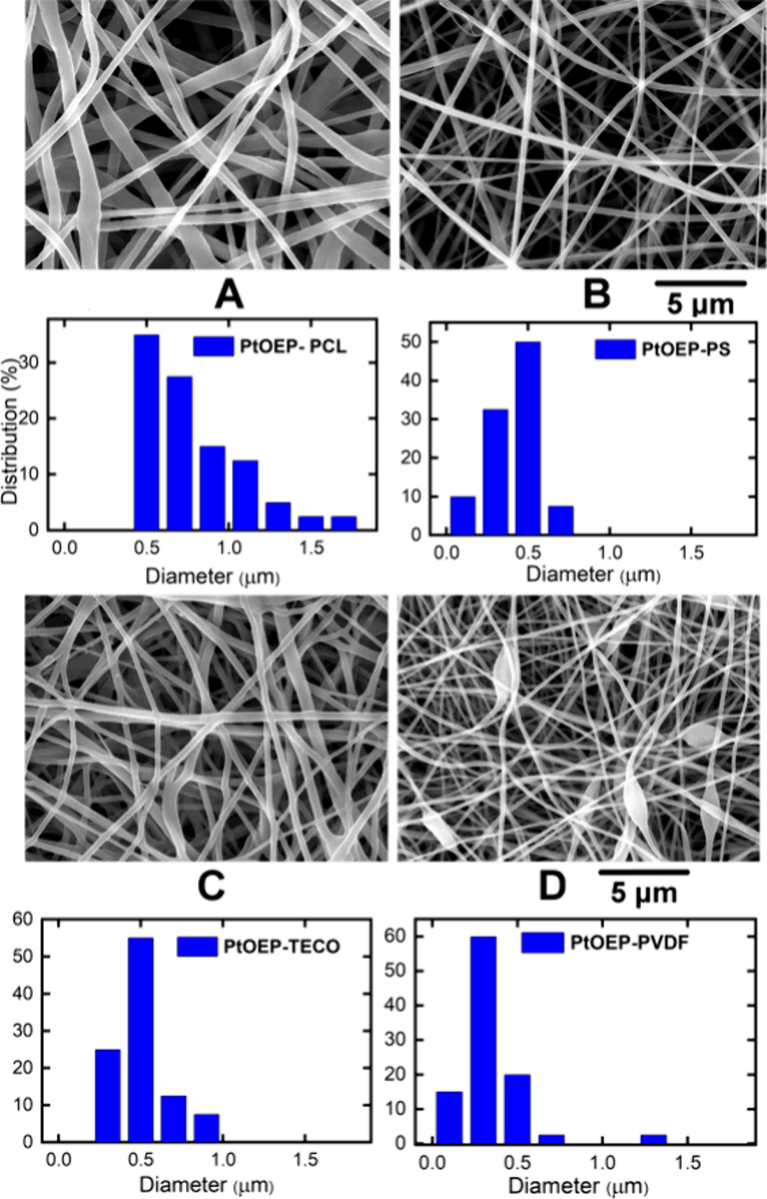
SEM micrographs of nanofiber materials: **PtOEP-PCL** (A), **PtOEP-PS** (B), **PtOEP-TECO** (C), and **PtOEP-PVDFHFP** (D) and corresponding histograms of nanofiber diameter distributions.

Figure 2 displays the surface morphology of the four prepared electrospun nanofiber sensors analyzed via SEM. The average diameters of the nanofibers are 760 ± 300, 390 ± 150, 470 ± 170, and 310 ± 180 nm for **PtOEP-PCL**, **PtOEP-PS**, **PtOEP-TECO,** and **PtOEP-PVDFHFP,** respectively. The basic material and spectral characteristics, including the diameter of the prepared nanofibers, the area density and thickness of the nanofiber membranes, the absorption (λ_abs_), and the luminescence maximum (λ_L_) of PtOEP in different polymeric nanofibers, are listed in Table 1.

**Table 1:** Basic Characteristics of the Prepared Nanofiber Membranes with Encapsulated Sensor Molecules PtOEP (1 wt %)

Material	Nanofiber diameter (nm)	Area density (g/m^2^)	Material thickness (μm)	λ_abs_ (nm)	λ_L_ (nm)
**PtOEP- PCL**	760 ± 300	4–20	20	381	645
**PtOEP-TECO**	390 ± 150	1–5	10	380	645
**PtOEP- PS**	470 ± 170	6–11	130	383	645
**PtOEP-PVDFHFP**	310 ± 180	3–9	10	377	646

### Photophysical Processes

The basic photophysical processes after the absorption of light by an oxygen sensor are illustrated in Figure 1B. Luminescent triplet states of PtOEP formed after absorption of light by PtOEP (with absorption and luminescence spectra in Figure 1C, D) are effectively quenched by ground-state oxygen to reduce the intensity of their red luminescence.

The luminescence kinetics of all the materials exhibited deviations from single exponential decay, and they were fitted by a double exponential function. The average lifetime of the luminescence decay was calculated as τ_*L*_*= (A*_*1*_τ_*1*_*+A*_*2*_τ_*2*_*)/(A*_*1*_*+A*_*2*_*)*, where *A*_*i*_ and τ_*i*_ represent the amplitudes and lifetimes of the double exponential process, respectively. The fraction of the long-lived triplet states responsible for luminescence trapped by oxygen in an oxygen atmosphere was also calculated as *F*_T_^O2^ = (τ_*L*0_ – τ_*L*O2_)/τ_*L*0_, where, τ_*L0*_ and τ_*L*O2_ are the luminescence lifetimes in vacuum and oxygen, respectively. The bimolecular rate constant of the luminescence quenching by oxygen (*k*_q_) was calculated using Stern–Volmer equation adapted for time-resolved measurements:
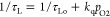
1where, *p*_O2_ is the oxygen pressure. Table 2 summarizes important parameters characterizing the luminescence lifetime of PtOEP in several polymer nanofibers without oxygen (τ_L0_) as well as its sensitivity to oxygen quenching (*F*_T_^O2^, *k*_*q*_). These characteristics are influenced primarily by the oxygen diffusion coefficient of the polymer used.

**Table 2:** Photophysical/Luminescence Parameters of 1 wt % PtOEP Encapsulated in Different Polymer Nanofiber Membranes

	τ_L_ (μs)^a^				
Material	oxygen	air	vacuum	*F*_T_^O2^	*k*_*q*_ s^–1^Torr^–1^
**PtOEP- PCL**	1.41	6.20	60.5	0.98	942
**PtOEP-PS**	4.46	15.2	83.9	0.95	284
**PtOEP-TECO**	3.20	11.1	80.8	0.86	408
**PtOEP-PVDFHFP**	11.2	62.0	81.4	0.86	111

a error less than 2%

The values of *k*_q_ = 942 s^–1^ Torr^–1^ and *F*_T_^O2^ = 0.98 for **PtOEP-PCL** are higher than those for similar **PtOEP-PS**, **PtOEP-TECO,** and **PtOEP-PVDFHFP** membranes. The high value of the luminescence lifetime in the absence of oxygen (τ_L0_ ∼ 60.5 μs) and the significant changes in the luminescence kinetics (lifetime) with increasing oxygen content led to corresponding changes in luminescence intensity (Figure S1 in the Supporting Information).

### Sensing Performance of Nanofiber Materials

The PtOEP luminescence of four different polymeric nanofiber materials by oxygen was monitored by steady-state luminescence, and the oxygen content was alternatively determined by the Clark electrode. The results were evaluated using Stern–Volmer dependence for luminescence quenching by oxygen:
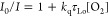
2where, *I*_0_ and *I* are the luminescence intensities without and with the oxygen quencher, [O_2_] is the oxygen concentration, *k*_q_ is the bimolecular rate constant of quenching, and τ_0_ is the lifetime of the luminescent excited state without oxygen.

**Figure 3:**
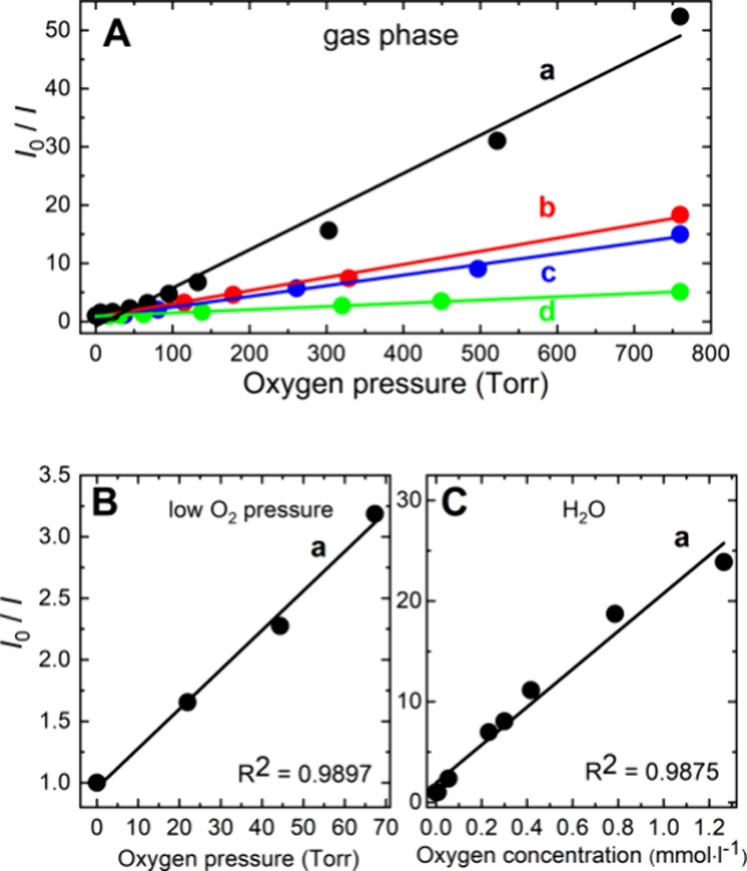
Stern–Volmer quenching of PtOEP luminescence (eq 2) in **PtOEP-PCL** (a), **PtOEP-PS** (b), **PtOEP-TECO** (c), and **PtOEP-PVDFHFP** (d) by oxygen in the gas phase (A) with corresponding zoom for low oxygen pressure (B), and in water (C).

Critical sensing parameters such as the fraction of triplet states quenched by oxygen (F_T_^O2^), bimolecular quenching constants of the triplet states by oxygen (*k*_*q*_), and the *I*_*0*_*/I* ratio are most appropriate for **PtOEP-PCL** (Table 2 and Figure 3).

**PtOEP-PCL,** with favorable photophysical properties and sensing properties in a gas atmosphere (Figure 3A,B), was also studied in detail in aqueous media (Figure 3C). As illustrated, the material in both phases exhibited high sensitivity. The *I*_0_/*I* ratios in oxygen-free water (*I*_0_) and water saturated with oxygen (*I*_100_) and in an oxygen atmosphere reached 25.8 and 52.4, respectively. The Stern–Volmer plots exhibited small deviations from linear interpolation: R^2^ = 0.989 for **PtOEP-PCL** in the gas phase (dynamic range 0.8% - 100%, i.e. 6.23–760 Torr), R^2^ = 0.989 for **PtOEP-PCL** at low concentration/pressure of oxygen (dynamic range 0.8% - 9%, i.e. 6.23–67 Torr) and R^2^ = 0.988 for **PtOEP-PCL** in water (dynamic range 0.6% - 100% saturation, i.e. 0.007–1.264 mmol/L). The estimated errors calculated from the coefficient of determination (R^2^), variance of data, and total number of experimental points were ca. 4–5% for both gas phase and aqueous media.

### Reversibility and Photostability

**Figure 4:**
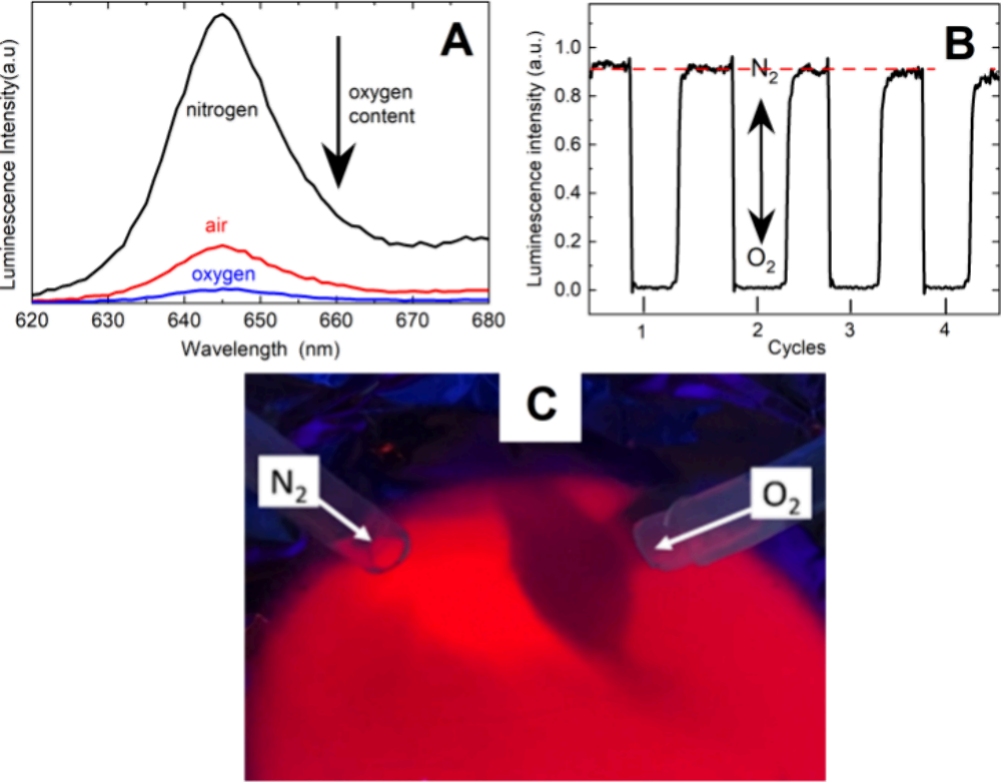
Changes in the luminescence spectra of the **PtOEP-PCL** sensor in nitrogen, air, and oxygen atmospheres (A) and after several cycles of filling the cell with nitrogen (high luminescence intensity) and oxygen (low intensity) (B). Image of the sensor luminescence under UV light with flow of N_2_ (luminescence amplification) and O_2_ (luminescence quenching) (C).

The **PtOEP-PCL** material was fixed on quartz glass and irradiated by light. The luminescence intensity was measured during several cycles of saturation with nitrogen (high luminescence intensity) and oxygen (low intensity) (Figure 4A, C). The luminescence intensity remained at the same level during all four nitrogen–oxygen cycles (Figure 4B).

The extended photostability tests (Figure S2 in the Supporting Information) indicated that the photodegradation of the PtOEP during irradiation was moderate but still acceptable because the excitation time for obtaining a reasonable luminescence signal from the sensor was on the scale of a few seconds.

### Response and Recovery Times

The response and recovery times are defined as the time required for a 95% change in the total luminescence intensity after the exchange of nitrogen with oxygen and oxygen with a nitrogen atmosphere, respectively (Figure S3 in the Supporting Information). A fast response and recovery time of less than 1 s were calculated from three independent measurements for all the materials (Table 3). Both the response and recovery times are comparable with the values published by Xu et al.^8^ for core–shell nanofibers consisting of poly(ether sulfone) with PCL (0.24/0.39 s), considering that the response and recovery times also include the time required to exchange gases.

**Table 3:** Sensitivity (Maximum *I*_0_/*I*_ratio_), Response, and Recovery Time for 1 wt % PtOEP Dispersed in Different Polymer Nanofiber Materials

Material	I_0_/I	Response time (s)	Recovery time (s)
**PtOEP-PCL**	52.4	0.37 ± 0.03	0.58 ± 0.20
**PtOEP-PS**	18.3	0.32 ± 0.03	0.55 ± 0.05
**PtOEP-TECO**	15.0	0.35 ± 0.05	0.53 ± 0.19
**PtOEP-PVDFHFP**	5.1	0.25 ± 0.05	0.37 ± 0.13

### Confocal Luminescence Microscopy

We used confocal luminescence microscopy to assess the homogeneity of the sensor materials, namely to verify whether PtOEP luminescence kinetics are comparable at different submicrometer locations within the nanofiber material.

**Figure 5:**
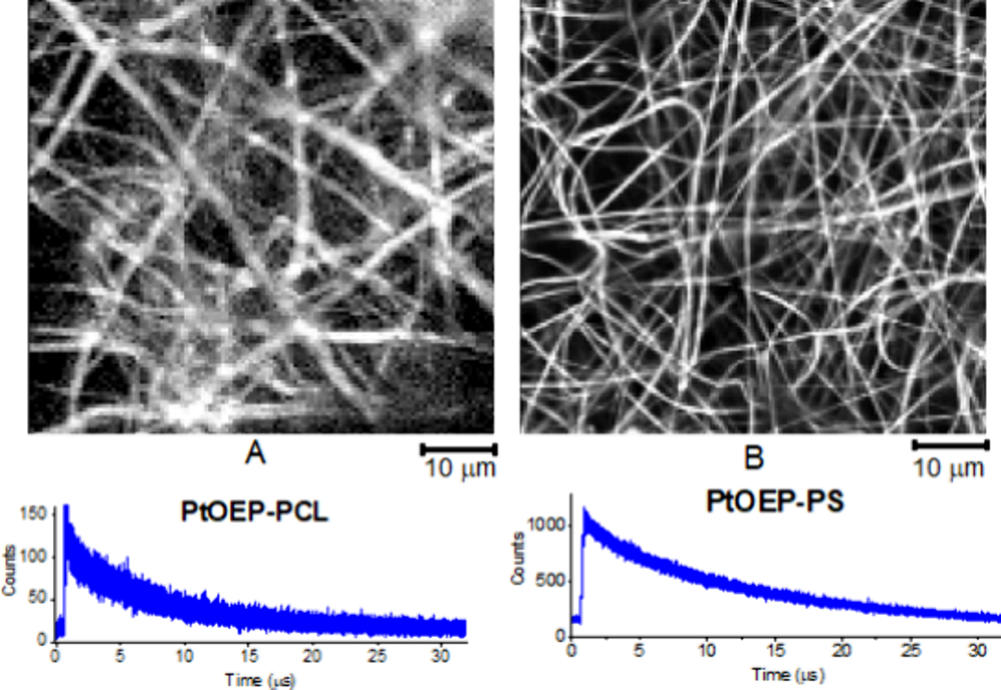
Confocal luminescence intensity images of **PtOEP-PCL** (A) and **PtOEP-PS** (B) with the corresponding kinetics of luminescence decay.

Confocal luminescence intensity images of both **PtOEP-PCL** (Figure 5A) and **PtOEP-PS** (Figure 5B) show the nanofiber structure of the materials. For lifetime measurements, the excitation laser beam was focused at randomly selected regions of the nanofibers because accurate luminescence lifetime imaging, which is based on a calculation of kinetics for each pixel in the image, requires an extremely long acquisition time in comparison with the fluorescence lifetime imaging of similar materials containing free base porphyrins.^27^ The luminescence lifetimes of PtOEP measured at 10 different locations on **PtOEP-PCL** varied between 6.5 and 7.2 μs, with an average value of τ_L_ = 6.8 ± 0.3 μs. In contrast, the lifetime τ_L_ reached 12.4 ± 0.1 μs for **PtOEP-PS,** reflecting differences in oxygen diffusion and the diameter of the nanofibers between both materials. Both lifetime values correspond with those obtained by time-resolved luminescence (Table 2), indicating similar diffusion of oxygen to all the PtOEP photosensitizer molecules encapsulated inside the individual materials fabricated from one polymer.

### Photogeneration of Singlet Oxygen

The quenching of PtOEP triplets encapsulated in polymeric materials and nanoparticles by oxygen in the ground state also leads to the formation of singlet oxygen, O_2_(^1^Δ_g_) (Figure 1B);^28^ the efficacy (quantum yield) of this process strongly depends on the concentration of oxygen. Direct measurement of the weak luminescence of O_2_(^1^Δ_g_) at 1270 nm revealed that the estimated lifetime of O_2_(^1^Δ_g_) in **PtOEP-PCL** (τ_Δ_) was approximately 10 μs (Figure S4 in the Supporting Information).

The iodide method was used to monitor O_2_(^1^Δ_g_) diffused outside nanofibers, where it could photooxidize bacteria and other biological targets, whereas the direct method based on NIR luminescence) predominantly reflects its properties (decay kinetics) inside nanofibers^20^ with a limited photooxidation/antimicrobial potential of O_2_(^1^Δ_g_).

**Figure 6:**
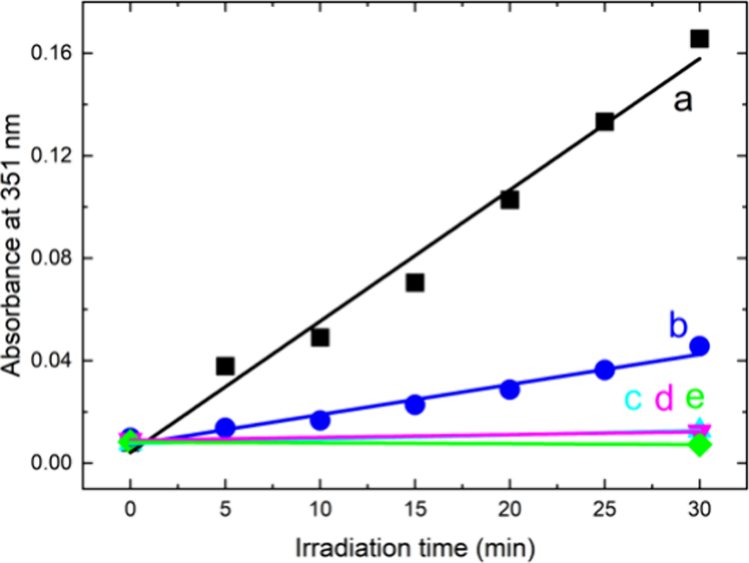
Time course of the absorbance of I_3_^–^ at 351 nm formed by photooxidation of the iodide detection solution with O_2_(^1^Δ_g_) during continuous irradiation of **PtOEP-PCL** by a 400 W solar simulator without (a) and with a 0.01 M NaN_3_ quencher (b). Blank experiments: **PtOEP-PCL** in iodide detection solution without (c) and with NaN_3_ (d) kept in the dark. Irradiated detection solution without any sample (e).

The iodide method^25^ is based on selective oxidation of iodide to I_3_^–^ by O_2_(^1^Δ_g_). The irradiation of **PtOEP-PCL** in the presence of iodide led to the formation of I_3_^–^, as shown by the gradually increasing absorbance at 351 nm during irradiation (Figure 6), in contrast to the behavior in the dark. The formation of I_3_^–^ is quenched by NaN_3_ (Figure 6B), a known physical quencher of O_2_(^1^Δ_g_).^29^ The experiments confirmed the photoproduction of O_2_(^1^Δ_g_) by VIS irradiation of **PtOEP-PCL** on its surface.

The slopes of the roughly linear kinetics of I_3_^–^ generation from irradiated **PtOEP-PCL** were compared with the slopes of irradiated **PtOEP-PS**, **PtOEP-TECO,** and **PtOEP-PVDFHFP** and 1 wt % tetraphenylporphyrin photosensitizer (TPP) in PCL (**TPP-PCL)** as a positive control (Figure S5 in the Supporting Information). Note that the slope of the linear kinetics of I_3_^–^ generation is proportional to the quantum yield of O_2_(^1^Δ_g_) and can be used for its estimation when comparing samples/standards with the same adsorption properties. As the samples have different absorbance spectra, the photogeneration of I_3_^–^ detected via its absorbance at 351 nm was corrected to the absorbance of the sample at the excitation wavelength (λ= 414 nm) using the absorption factor (1–10^–*A*^). Some deviation in the linearity of kinetics observed at the beginning of photooxidation is attributed to the adsorption of I_3_^–^ on the nanofiber membrane.

As illustrated in Figure S5 (Supporting Information), samples **PtOEP-PCL, PtOEP-PS** and **PtOEP-PVDFHFP** exhibit roughly the same kinetics of I_3_^–^ generation in contrast to **PtOEP-TECO**, which has a lower rate of photooxidation, is probably attributed to partial aggregation of PtOEP in the Tecophilic polymer, resulting in quenching of its excited states and O_2_(^1^Δ_g_).

In contrast, **TPP-PCL** has a more efficient photooxidation rate, which corresponds with the fact that TPP has a high quantum yield of O_2_(^1^Δ_g_) (Φ_Δ_∼ 0.62)^30^ and therefore has an enhanced photooxidation rate compared with PtOEP in the same PCL matrix.

### Photoantibacterial Effect of the PtOEP-PCL Sensor

In previous studies,^20,31^ it was reported that nanofiber membranes with tetraphenylporphyrin photosensitizer and strong antibacterial behavior. Tetraphenylporphyrin does not exhibit luminescence from the triplet states and cannot be applied for oxygen sensing; most of the energy is used for the formation of antibacterial O_2_(^1^Δ_g_) with very high quantum yield (typically more than 0.5). In contrast, luminescence channel of PtOEP significantly reduces the quantum yield of O_2_(^1^Δ_g_) depending on oxygen concentration down to 0.24.^28^ The antibacterial character of the **PtOEP-PCL** sensor was verified via two tests (see the Experimental section for details) to evaluate the antibacterial ability of the sensor surfaces.

**Figure 7:**
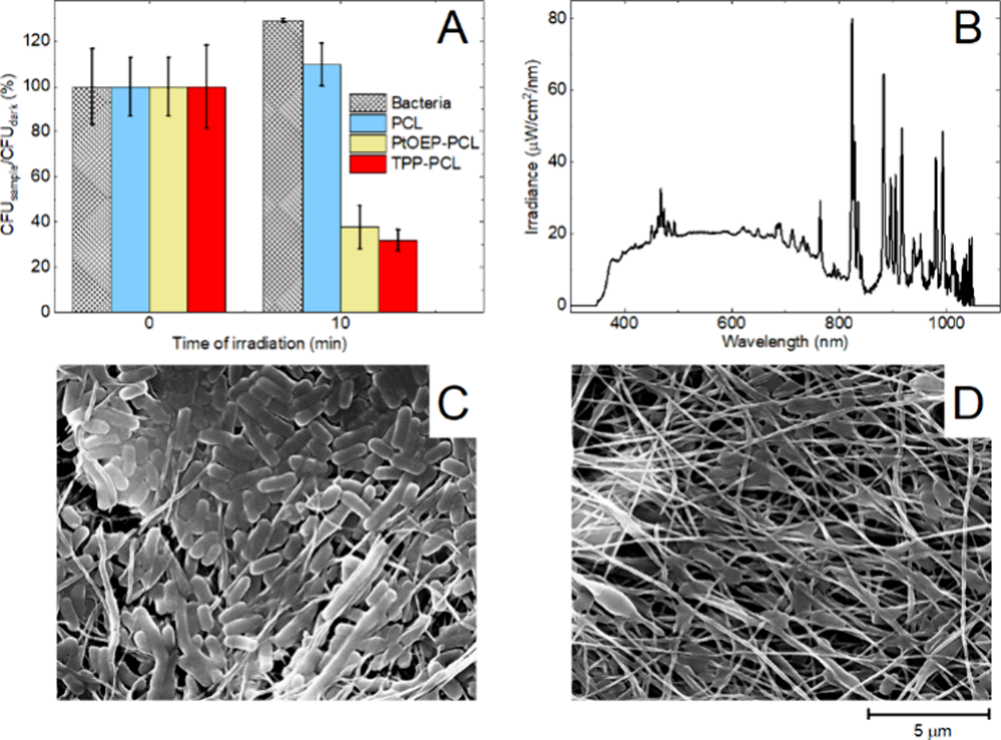
Antibacterial activity of **PtOEP-PCL** was evaluated by comparing the number of surviving colony-forming units (CFUs) of *Escherichia coli* on agar plates. The agar plates were inoculated with bacteria (positive control) and bacteria obtained from **PCL**, **TPP-PCL**, and **PtOEP-PCL** samples, which were irradiated with bacteria for 10 min or kept in the dark for the same time as the dark controls. The resulting CFU counts and corresponding statistics are obtained from three independent experiments (A). The emission spectrum of the solar simulator used for irradiation (B). SEM micrographs of **PtOEP-PCL** nanofiber membrane inoculated with bacteria and kept in the dark (C), and illuminated with a solar simulator for 10 min after inoculation (D) both after 48 h of incubation, demonstrating significant reduction of a biofilm formation.

The quantitative CFU counting antibacterial test (Figure 7A) was based on the irradiation of inoculated bacteria on the sensor surface with the next removal (shaking up) of bacteria from the sensor surface to the cultivation media after irradiation/dark conditions. This test revealed that **PtOEP-PCL** is a slightly less efficient photoproducer of O_2_(^1^Δ_g_) and light-induced antibacterial material than **TPP-PCL** is however, **PtOEP-PCL** can still inactivate more than 70% of bacteria after 10 min of irradiation by a solar simulator (Figure 7B). Representative photos of agar plates in dark/irradiated conditions are shown in Figure S6 in Supporting Information.

The qualitative antibacterial test, which was based on direct irradiation of inoculated bacteria on the sensor surface (Figure S7 in the Supporting Information), revealed a strong surface sterilization effect. For better visualization of bacterial colonies (blue-green color) on the sensor surface, the *E. coli* strain DH5α with the pGEM11Z plasmid, which produces β-galactosidase, was used together with X-gal (5-bromo-4-chloro-3-indolyl-β-D-galactopyranoside) as a β-galactosidase substrate in cultivation agar. The colonies producing β-galactosidase cleaved the X-gal substrate into an indolyl dye and were clearly visible on the sensor surface as green spots. Only the samples of inoculated PCL membranes doped with PtOEP (**PtOEP-PCL)** and irradiated for 10 min by a 400 W solar simulator exhibited antibacterial effects (almost no visible colonies) in contrast to the control samples, i.e., the samples without PtOEP or **PtOEP-PCL** kept in the dark (Figure S7 in the Supporting Information). Also, SEM images confirmed antibacterial effect/inhibition of biofilm formation toward bacteria inoculated on its surface when irradiated by visible light (Figure 7C, D). The steady-state luminescence of the sensor is reduced by approximately 8% after 48 h of bacterial incubation (formation of a biofilm) on its surface (Figure S8 in the Supporting Information). The antibacterial effect of irradiated **PtOEP-PCL** was similar to that of **TPP-PCL**, which served as a reference control.

Note that TPP in polymeric matrices and nanoparticles prepared from nanofibers are frequently used in the photodynamic inactivation of pathogens due to their simple preparation, photostability, and high quantum yield of O_2_(^1^Δ_g_),^32^ and short-lived O_2_(^1^Δ_g_) with a diffusion length of a few hundred nanometers can kill bacteria exclusively captured on the surface of the sensor. Any antibacterial application requires nanofiber membranes with a suitable surface for the effective detention of bacteria (or other pathogens) with a size comparable to the diffusion length of O_2_(^1^Δ_g_). The short diffusion pathway of O_2_(^1^Δ_g_) (tens to hundreds nm) even enables the efficient inactivation of pathogenic bacterial strains localized on human skin and/or close to nanofiber material without causing tissue damage.^33^

The photogeneration of O_2_(^1^Δ_g_) from the sensor and subsequent bacterial inactivation/sterilization during oxygen sensing can be applied in many biomedical fields, *e.g.*, for *in situ* sterilization of luminescent sensors in tissue engineering.^34,35^

## Conclusions

Platinum(II) octaethylporphyrin dispersed in polycaprolactone nanofibers represents a cheap, easy-to-prepare, and nontoxic oxygen sensor with a fast, very sensitive, reversible luminescent response and linear Stern–Volmer quenching behavior over the whole range of oxygen contents in both the gas atmosphere and aqueous media. Owing to the photogeneration of O_2_(^1^Δ_g_), the sensor also exhibited high surface antibacterial properties. Both the oxygen sensing ability and photogeneration of O_2_(^1^Δ_g_), which has antibacterial properties, benefit from the high oxygen permeability/diffusion coefficient of the PCL nanofiber matrix. The light-triggered self-sterilization effect can help, e.g., avoid the formation of a biofilm on the sensor surface. The sensor can be applied in systems where the dimensions are too small to use standard oxygen electrodes and/or where chemical probes are too toxic or are sensitive to the surroundings. The basic concept of oxygen-sensing and photosensitizing nanofiber membranes may contribute to the development of very sensitive, fast, and self-sterilizing luminescence sensors.

## Supporting Information Available

Luminescence and photodegradation of sensors, calculation of response and recovery times, photooxidation and antibacterial experiments (PDF)

## Abbreviations

PtOEP: platinum(II) complex of octaethylporphyrin
**PtOEP-PCL**: electrospun poly(ε-caprolactone) nanofiber material doped with PtOEP
**PtOEP-PS**: electrospun polystyrene nanofiber material doped with PtOEP
**PtOEP-PVDFHFP**: electrospun poly(vinylidene fluoride-*co*-hexafluoropropylene) nanofiber material doped with PtOEP
**PtOEP-TECO**: electrospun Tecophilic nanofiber material doped with PtOEP
