# Supplementary material for: Luminescent Oxygen Sensor with Self-Sterilization Properties Based on Platinum(II)octaethylporphyrin in Polymeric Nanofibers
Source: ACS Mater Au. 2025 Jan 1;5(2):331–8. doi: 10.1021/acsmaterialsau.4c00137 (PMC11907296; doi:10.1021/acsmaterialsau.4c00137)
Supplement: Supplementary file 1 — mg4c00137_si_001.pdf [file mg4c00137_si_001.pdf]

Supporting Information

Luminescent Oxygen Sensor with Self-Sterilization Properties based on Platinum(II)octaethylporphyrin in Polymeric Nanofibers

Pavel Ludačka,[a] Vojtěch Liška,[a] Jan Sýkora,[b] Pavel Kubát[b] and Jiří Mosinger[a]*

[a]*Faculty of Science, Charles University, Hlavova 2030, 128 43 Prague 2, Czech Republic*

[b]*J. Heyrovský Institute of Physical Chemistry of the Czech Academy of Sciences, Dolejškova 3, 182 23 Prague 8, Czech Republic*

Content

**Fig.S1**	Kinetics of **PtOEP-PCL** luminescence………………………………	**S-2**
**Fig.S2**	Photodegradation of **PtOEP -PCL** sensor ……………………………	**S-2**
**Fig.S3**	The response and the recovery time…………………………………...	**S-3**
**Fig.S4**	Decay of luminescence of singlet oxygen……………………………..	**S-4**
**Fig.S5**	The photooxidation test………………………………………………..	**S-4**
**Fig.S6**	The antibacterial effect of **PtOEP-PCL** visualized on agar plates…...	**S-5**
**Fig.S7**	The antibacterial effect of **PtOEP-PCL** evaluated by X-Gal method...	**S-5**
**Fig.S8**	Luminescence of **PtOEP-PCL** with/without bacterial contamination..	**S-6**

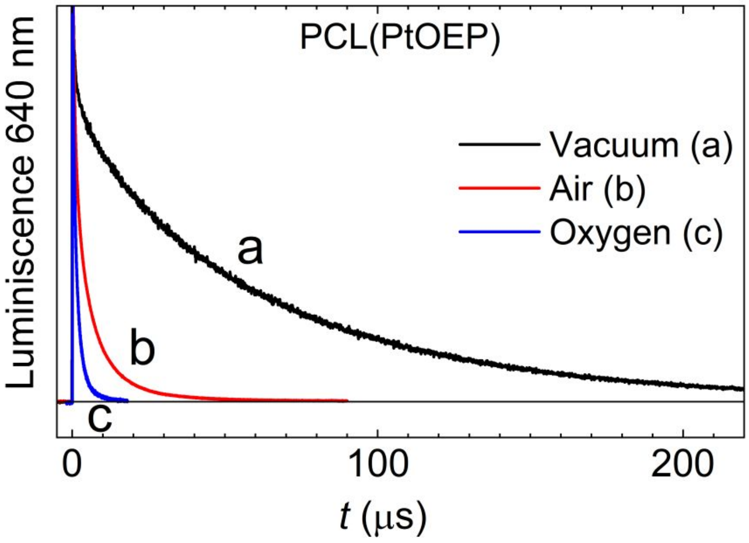

Figure S1. Kinetics of **PtOEP-PCL** phosphorescence in vacuum (a), air (b), and oxygen atmosphere (c).

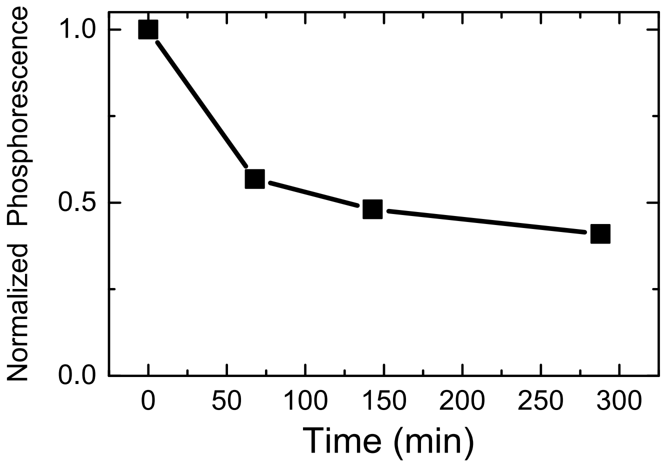

Figure S2. Photodegradation of **PtOEP-PCL** sensor during continuous irradiation at λ_{ex} = 385 nm (29 μW/cm^2).

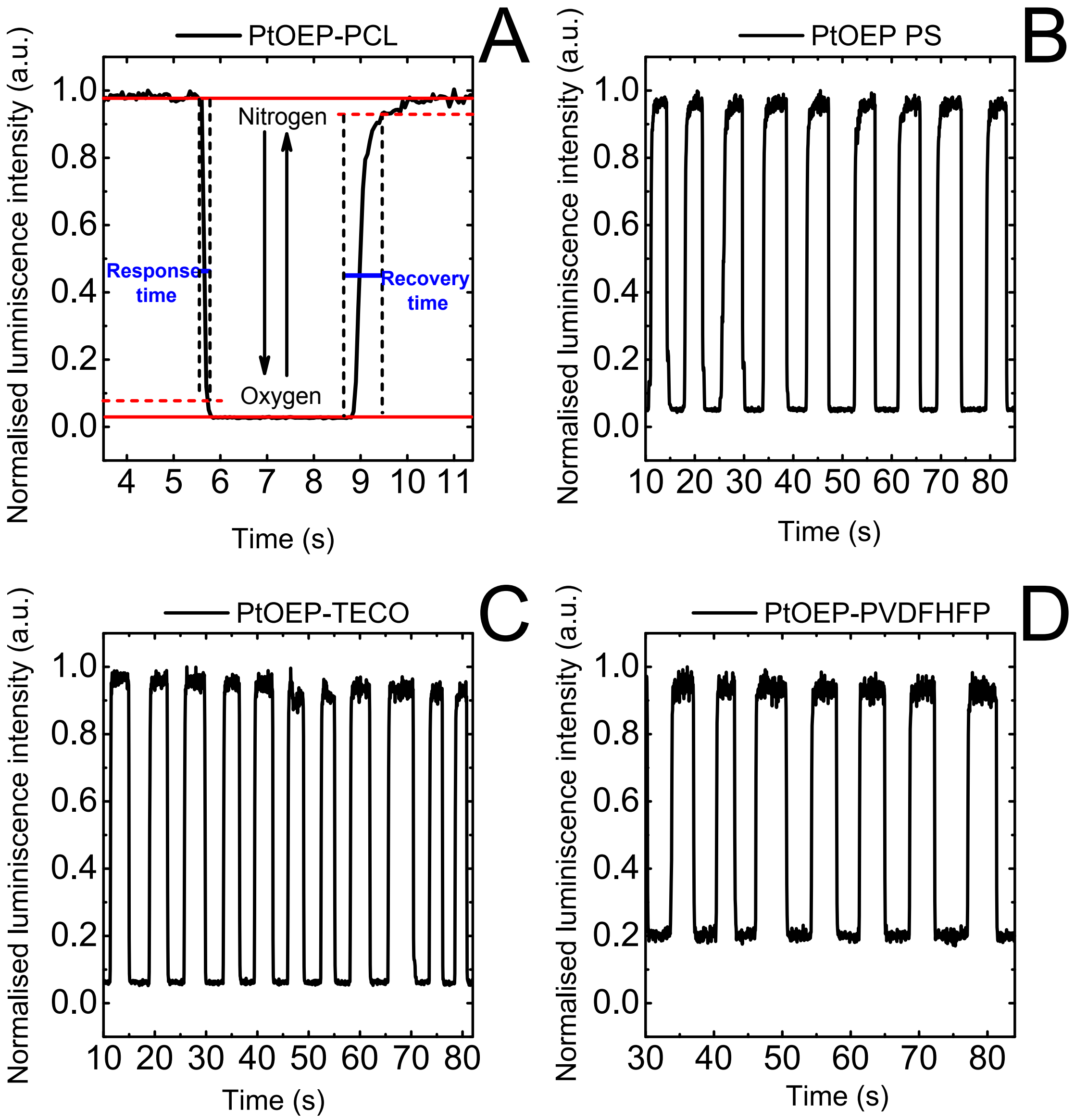

Figure S3. Illustration of measuring of the response and the recovery time measured for **PtOEP-PCL**, where red line denotes 95% of signal maximum/ minimum (A). Demonstration of reversibility of other tested materials **PtOEP-PS** (B), **PtOEP-TECO** (C), **PtOEP-PVHDHFP** (D).

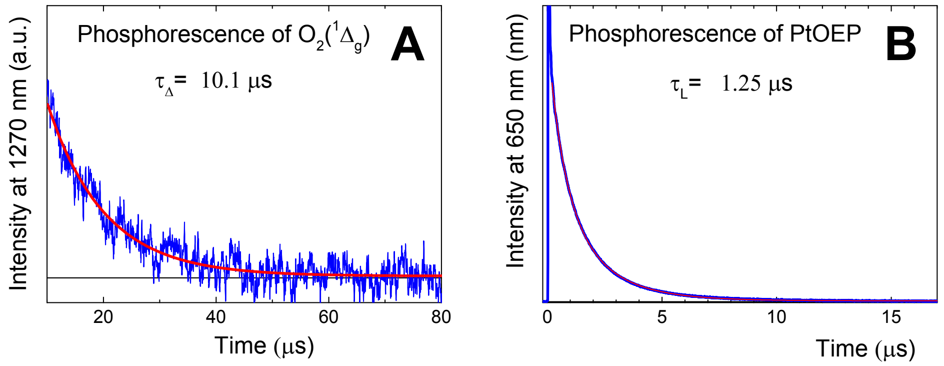

Figure S4. Decay of luminescence of $O_2(^1\Delta_g)$ at 1270 nm between 10 - 80 µs after excitation. (**A**), where the contribution of light scattering and phosphorescence of PtOEP can be neglected compared with short-lived luminescence of PtOEP in **PtOEP-PCL** (**B**). The lifetimes were calculated as single exponential fits to experimental data (red lines). Excited by Nd-YAG laser (355 nm, 5 ns)

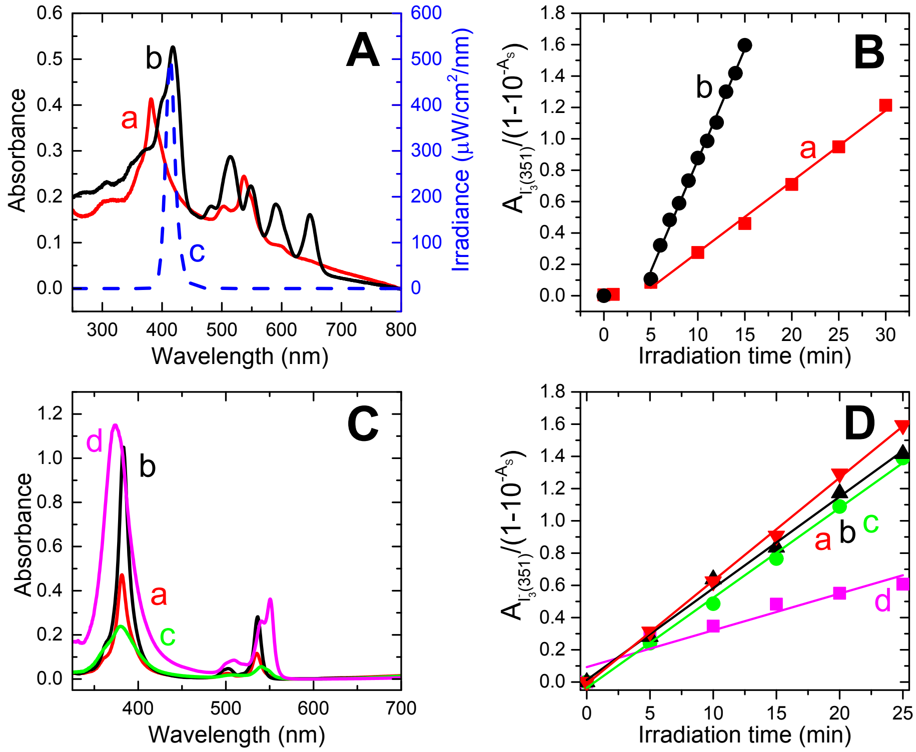

Figure S5. A: Absorbance of **PtOEP-PCL** (a) and **TPP-PCL** (b) and irradiance of LED source (λ_{ex} = 414 nm; c). **B:** The time course of absorbance of I_3^- at 351 nm formed by photooxidation of iodide detection solution with $O_2(^1\Delta_g)$ during continuous irradiation of **PtOEP-PCL** (a) and **TPP-PCL** (b). **C:** Absorbance of **PtOEP-PCL** (a), **PtOEP-PS** (b), **PtOEP-PVDFHFP** (c), **PtOEP-TECO** (d) and irradiance of LED source (λ_{ex} = 414 nm; e). **D:** The kinetics of I_3^- generation corrected to absorbance of the sample (A_s) at excitation wavelength (λ_{ex} = 414 nm). **PtOEP-PCL** (a); **PtOEP-PS** (b); **PtOEP-PVDFHFP** (c); **PtOEP-TECO** (d).

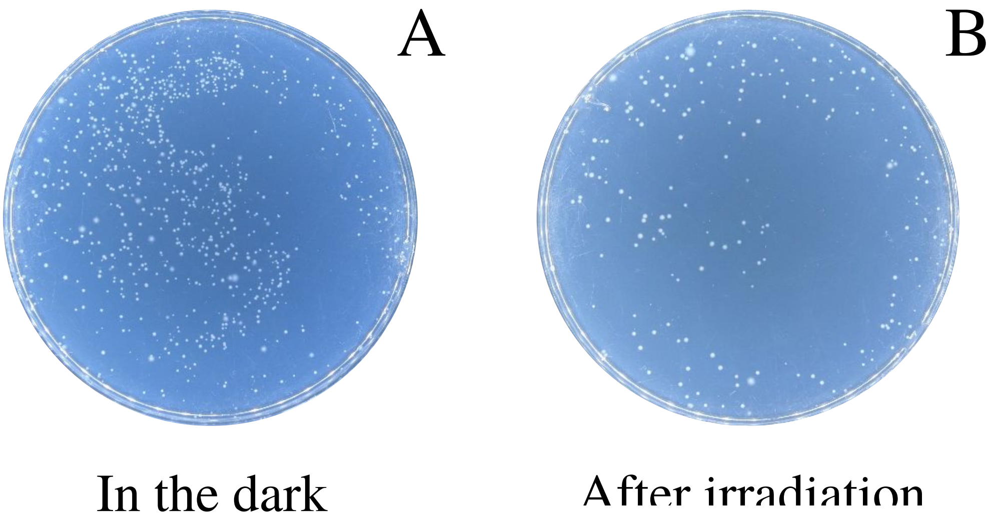

Figure S6. Figures A and B illustrate the colonies of *E. coli* on agar plates, which represent the outcome of the antibacterial effect of **PtOEP-PCL**. Figure A shows bacteria from media with the specimen after being incubated in darkness for 10 minutes, while Figure B shows bacteria from media with the specimen after a 10-minute exposure to solar simulator.

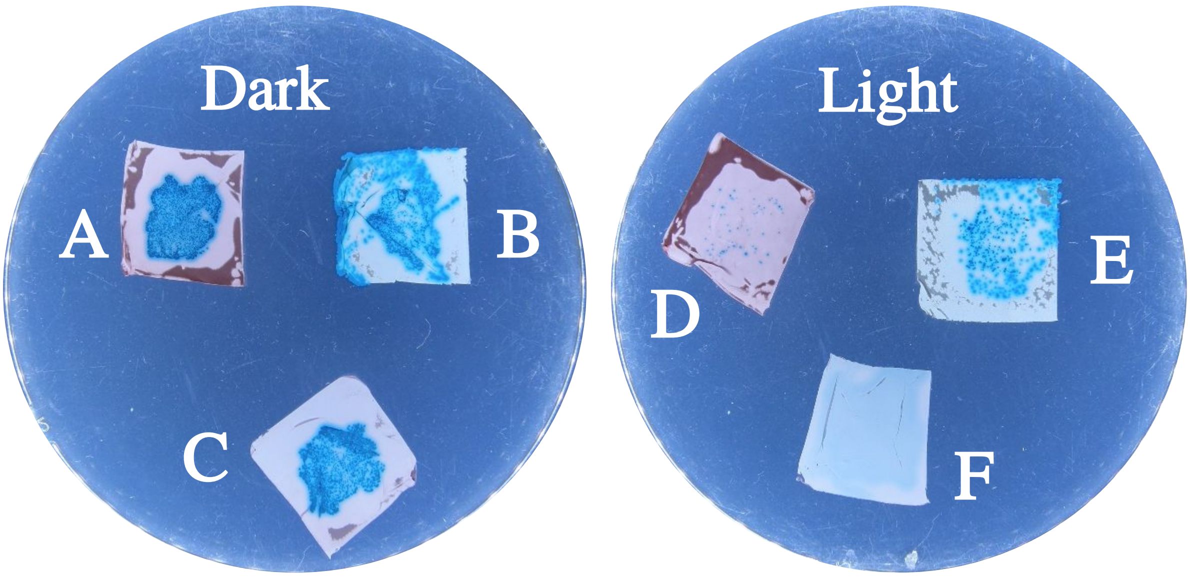

Figure S7. The images of the X-Gal test demonstrate the antibacterial properties of the **PtOEP-PCL** sensor. The blue dots represent surviving bacterial colonies, which are visible on all dark samples (A-C) and on the illuminated negative control of pure **PCL** membrane (E). No surviving bacteria are visible on the positive control of **TPP-PCL** (F) and on the **PtOEP-PCL** sensor sample (D). Samples were irradiated for 10 minutes with a 400 W solar simulator.

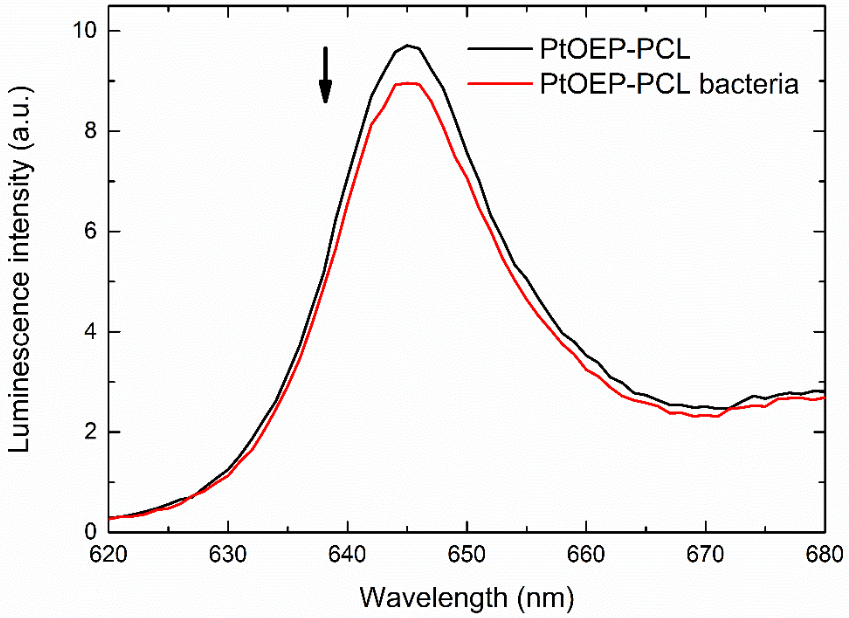

Figure S8. Luminescence emission spectra of PtOEP-PCL samples recorded after 2 days of incubation in pure culture medium (black line) and in culture medium inoculated with *Escherichia coli* (red line). The black arrow indicates an 8% decrease in luminescence. To evaluate the effect of biofilm formation on luminescence intensity, 100 μL of a diluted *Escherichia coli* suspension or 100 μL of pure media was applied to the surface of PtOEP-PCL membranes (2.25 cm^2). The membranes were incubated in darkness at 37 ˚C for 48 hours to allow the bacteria to overgrow and develop a biofilm on the membrane surface. After incubation, the samples were analyzed using an Edinburgh Instruments FLS 980 spectrometer (Edinburgh, Great Britain) under identical measurement parameters to assess differences in steady-state luminescence intensity.
